# CDCP1 Deletion Protects Against Pressure Overload-Induced Cardiac Dysfunction and Fibrosis in Mice

**DOI:** 10.21203/rs.3.rs-9236741/v1

**Published:** 2026-05-08

**Authors:** Naveen Pereira, Rachad Ghazal, Akshatha N. Srinivas, Min Wang, Jenny Jia Ling Cao, Li Wang, Irene Marín-Goñi, William Sherman, August John, Hridyanshu Vyas, Carolyn Roos, Runqing Huang, Tamas Ordog, Chen Wang, Christy Trussoni, Do Young Lim, Darren Baker, Gianfranco Sinagra, Richard Weinshilboum, Duan Liu, Laura J. Lambert, Jordan Miller, Daniel Tschumperlin

**Affiliations:** Mayo Clinic; Mayo Clinic; Mayo Clinic; Mayo Clinic; Department of Cardiovascular Diseases, Mayo Clinic; Mayo Clinic; Mayo Clinic; Mayo Clinic; Mayo Clinic; Mayo Clinic; Mayo Clinic; Mayo Clinic; Mayo Clinic; Mayo Clinic; Mayo Clinic; Mayo Clinic; Mayo Clinic; Ospedali Riuniti and University of Trieste; Mayo Clinic; Mayo Clinic; Mayo Clinic; Mayo Clinic; Mayo Clinic

**Keywords:** cardiac fibrosis, CDCP1, heart failure, mouse model, spatial transcriptomics

## Abstract

Human genomic studies link reduced CUB domain-containing protein 1 (CDCP1) expression with myocardial recovery in heart failure. While CDCP1 regulates cardiac fibroblast proliferation in vitro, it’s in vivo role in cardiac fibrosis remains unclear. Using a *Cdcp1*-knockout (KO) angiotensin II/phenylephrine mouse model, we show that Cdcp1 deletion reduces echocardiographic left ventricular mass, histologic cardiac fibrosis, and pro-fibrotic gene expression, along with decreased fibroblast activation and inflammatory markers. Spatial transcriptomics identified a pressure overload–expanded fibroblast subpopulation enriched for growth factor and TGF-β signaling (FB5), which was markedly attenuated in *Cdcp1*-KO hearts, alongside reduction of a pro-inflammatory cardiomyocyte subtype (CM4). Complementary studies in human ventricular fibroblasts demonstrate that *CDCP1* knockdown reduced extracellular matrix gene expression and collagen I deposition. These findings establish CDCP1 as a regulator of cardiac fibrotic remodeling in vivo and open avenues for its further investigation as a potential therapeutic target.

## INTRODUCTION

Cardiac fibrosis is a hallmark pathological feature in various cardiovascular diseases and a major driver of heart failure progression.^1–3^ This process is characterized by excessive extracellular matrix (ECM) accumulation that is mediated by activated cardiac fibroblasts,^4^ and contributes significantly to myocardial stiffness and dysfunction. Despite its clinical significance, effective therapies targeting cardiac fibrosis remain limited.^5,6^ Chronic pressure overload induces left ventricular (LV) hypertrophy and progressive interstitial ECM accumulation.^7,8^ This excessive collagen deposition elevates filling pressures, compromises chamber compliance, and predisposes to arrhythmias and heart failure progression.^9^ Mechanistically, cardiac fibrosis involves resident cardiac fibroblast proliferation, migration and transdifferentiation into α-smooth muscle actin (αSMA)-expressing myofibroblasts, which secrete collagens resulting in adverse cardiac remodeling.^10^ Current antifibrotic therapies with demonstrated clinical efficacy remain elusive,^11,12^ stressing the need to identify novel molecular targets.

We previously performed a genome-wide association study (GWAS) for myocardial recovery in patients with recent onset heart failure.^11,12^ That GWAS identified a genetic locus (index SNP rs6773435) that is an expression quantitative trait locus (eQTL) for the *CUB domain-containing protein 1* (*CDCP1*) gene in cultured fibroblasts.^13^ Reduced *CDCP1* expression was associated with improved left ventricular ejection fraction (LVEF) in heart failure patients. We further demonstrated that *CDCP1* expression is upregulated in human cardiac fibroblasts in response to profibrotic stimuli and that its knockdown attenuates fibroblast proliferation.^11^ However, its role in cardiac fibrosis and remodeling *in vivo* remains undefined.

In this study, we investigated the role of CDCP1 in cardiac fibrosis using a pressure overload-induced murine model with *Cdcp1* gene knockout (KO). This model recapitulates the chronic neurohormonal stress milieu characteristic of heart failure, providing a biologically relevant context to functionally interrogate CDCP1 in cardiac fibrosis. *Cdcp1*-KO mice demonstrated attenuated pathological remodeling in response to chronic angiotensin II/phenylephrine (AngII/PE) infusion, preserved cardiac function, and reduced fibrotic burden compared to wild-type (WT) controls. Transcriptomic analysis revealed that *Cdcp1* deletion suppresses profibrotic and proinflammatory gene networks. Spatial transcriptomics revealed that *Cdcp1* deletion induces region-specific alterations in fibrogenic gene signatures within the myocardium, including reduced inflammation and extracellular matrix organization associated cardiomyocyte subpopulations and attenuation of growth factor and TGF-β signaling associated fibroblast subtypes. Our findings establish *Cdcp1* as a critical mediator of cardiac fibrosis, providing the direct functional evidence of its role in cardiac pathophysiology and supporting its potential as a therapeutic target for cardiac fibrosis.

## METHODS

### Animal Model and Ethical Approval

All procedures conformed to the Guide for the Care and Use of Laboratory Animals (U.S. Department of Health and Human Services) and were approved by the Institutional Animal Care and Use Committee (IACUC) of the Mayo Clinic. Mice on an FVB/NJ background (The Jackson Laboratory, Bar Harbor, ME) were housed under specific pathogen-free (SPF) conditions with controlled temperature (22 ± 1°C) and humidity (55 ± 5%), on a 12-h light/dark cycle. Standard rodent chow and water were provided *ad libitum*. Throughout the study, all efforts were made to minimize discomfort during procedures such as ear tagging, tissue sampling for genotyping, and anesthesia for osmotic pump implantation.

### Generation of Cdcp1 Knockout Mice

*Cdcp1*-null mice were generated on an FVB/NJ genetic background by CRISPR-Cas9 editing to remove exon 1 of *Cdcp1* gene. Four guide RNAs were designed to flank exon 1: 5g-1 (5’-GTAGATGGTCTGGGACCTCG-3’), 5g-2 (5’-GGGGGGGTCATCACAACATG-3’), 3g-1 (5’-GGGATACTCGATTGGGACGT-3’), and 3g-2 (5’-GAGAACGTCCTCCTAAGGCT-3’). Genotyping was performed using primers DY164 (forward: 5’-GCATGGGCTTCTGTTTCTGT-3’) and DY165 (reverse: 5’-GCACGGACAGCTAAAATGGT-3’). Founder mice harboring exon deletion confirmed by PCR genotyping of ear DNA were bred to homozygosity to obtain *Cdcp1*^*−/−*^ (KO) offspring and littermate-matched WT controls. Both male and female mice, aged 8–12 weeks, were used in all experiments. Mice were randomly assigned to saline or AngII/PE treatment groups, with males and females distributed as evenly as possible across genotypes and treatment conditions. Mice were monitored daily for overall health, body weight, and signs of distress.

### Angiotensin II/Phenylephrine Infusion Model

To induce pressure overload and cardiac fibrosis, osmotic minipumps (ALZET^→^ model 1002, DURECT Corporation, Cupertino, CA) were implanted subcutaneously at 10 weeks of age under 1.5% isoflurane anesthesia. Minipumps were pre-loaded to deliver either saline (0.9% NaCl) as a control, or AngII (1.2 μg/g/day; Sigma-Aldrich, Cat. No. A9525, St. Louis, MO, USA) plus phenylephrine (PE) HCl (35 μg/g/day; Sigma-Aldrich, Cat. No. P6126–5G) for 28 days. Incisions were closed with a single 5 – 0 nylon suture, and mice were placed in warmed recovery chambers postoperatively. Four experimental cohorts were established: Saline_WT (WT mice receiving saline infusion), Saline_KO (*Cdcp1*^*−/−*^ mice receiving saline), AngII/PE_WT (WT mice receiving AngII/PE), and AngII/PE_KO (*Cdcp1*^*−/−*^ mice receiving AngII/PE). All animals were monitored for any adverse effects, including changes in mobility or grooming. No mortality or overt toxicity was observed in any group during the 4-week infusion period. A separate cohort of mice was monitored for 14 weeks to examine the effect of *Cdcp1* deletion on long-term survival during pressure overload. For all other endpoints (echocardiography, histology and transcriptomics), mice were euthanized at 4 weeks post-implantation.

### Echocardiographic Assessment of Cardiac Function

Transthoracic echocardiography (TTE) was performed at baseline and at 4 weeks post-minipump implantation using the Vevo^→^ F2 Imaging System (FUJIFILM VisualSonics, Toronto, Canada) equipped with a 46 – 20 MHz linear-array transducer. Mice were lightly anesthetized (1–2.5% isoflurane), positioned supine on a warming platform, and the heart rate was maintained between 450–600 bpm to minimize anesthesia-induced cardio depression through adjustment of inhaled isoflurane concentration. Two-dimensional (2D) and M-mode images were acquired in the parasternal short-axis view at the mid-papillary level. Left ventricular (LV) mass index (LVMI), LV ejection fraction (LVEF), and LV fractional shortening (LVFS) were calculated from M-mode measurements as previously described.^13^ All acquisitions and their analysis were conducted in a blinded fashion to the animal’s genotype and treatment group.

### Histopathology and Fibrosis Quantification

After the 28-day infusion period, mice were euthanized by CO_2_ inhalation, in accordance with approved IACUC protocol. Hearts were rapidly excised, rinsed in 10X Phosphate-Buffered Saline (PBS), and fixed in 10% neutral-buffered formalin at room temperature over 24 hours. Fixed tissues were embedded in paraffin and sectioned at 6 μm thickness. Collagen deposition was assessed histologically by Picrosirius Red staining (Polysciences Inc., Warrington, PA) following the manufacturer’s protocol. Light microscopy images were captured at 20X and 40X magnification (Nikon, Tokyo, Japan). Quantification of cardiac fibrosis was determined by analyzing Picrosirius Red-positive area in ≥ 5 randomly selected mid-myocardial fields per sample, excluding large epicardial vessels.

### Bulk-tissue RNA Sequencing

Snapfrozen LV tissues were pulverized under liquid nitrogen. Total RNA was extracted using Quick-RNA^™^ Miniprep Kit (Zymo Research, Cat. No. R2052). RNA integrity was assessed using an Agilent 2100 Bioanalyzer (Agilent Technologies, Santa Clara, CA, USA) and library preparation was carried out using the NEBNext^→^ Ultra^™^ II RNA Library Prep Kit for Illumina (New England Biolabs, Ipswich, MA, USA), following the manufacturer’s guidelines. Multiplexed libraries were loaded onto an Illumina NovaSeq^™^ 6000 flow cell (Illumina, San Diego, CA, USA) and sequenced in a 2·150 bp paired-end format. Base calling and raw data processing were managed by the NovaSeq Control Software (NCS). The raw reads were quality-checked, trimmed and aligned to Mus musculus reference genome (GRCm39: GCF_000001635.27) using STAR (v2.5.2b). Read counts were assigned to annotated genes with featureCounts (Subread v1.5.2) counting only the uniquely mapped reads in exonic regions. Differential expression analysis was performed using DESeq2 (R/Bioconductor) with the Wald test applied to obtain log_2_ fold changes and *P*-values. Genes with an adjusted *P*-value < 0.05 and an absolute log_2_ fold change > 1 were designated as differentially expressed. Functional gene set enrichment analysis (GSEA) was performed using the clusterProfiler R package on a ranked list of gene symbols ordered by log_2_ fold-change from DESeq2 differential expression.

### Spatial Transcriptomic Assays

Formalin-fixed, paraffin-embedded (FFPE) heart blocks were sectioned at 5 μm thickness. Tissues with RNA integrity number (RIN) > 6 were selected for spatial transcriptomics using the Visium Spatial Gene Expression platform (10x Genomics, Pleasanton, CA). Tissue sections with picrosirius red were placed on capture areas (≈5000 barcoded spots per 6.5·6.5 mm area, spot diameter: 55 μm, center-to-center distance: 100 μm), and imaged with a Leica Aperio VERSA (Leica Microsystems, Wetzlar, Germany) at 20X resolution. Permeabilization conditions were optimized according to the Tissue Optimization protocol (10x Genomics CG000238). Spot-based RNA capture, reverse transcription, cDNA amplification, and library construction were performed according to the Visium Spatial Gene Expression Slide & Reagent Kit (10x Genomics). Resultant libraries were sequenced on an Illumina NovaSeq^™^ 6000 system.

### Spatial Transcriptomic Data Analysis

Raw FASTQ files were processed to quantify spot-level gene expression. Spots on tissue were manually adjusted with Loupe browser (10x Genomics). The feature barcode expression matrices were analyzed and visualized in Seurat v5.1.0 (R/CRAN). Samples were normalized using sctransform method with additional log normalization and scaled based on gene counts. Harmony integration method was used for inter-sample comparison. Uniform Manifold Approximation and Projection (UMAP) was used for 2D visualization of clusters. Gene signatures distinguishing each cluster or subpopulation were identified using Seurat’s “FindMarkers” with a false discovery rate (FDR) < 0.05 and a log_2_ fold change threshold > 0.25. To characterize the cell-type proportions of spatial spots, Seurat’s integrated anchor-based deconvolution method was employed using previously published and annotated single-cell RNAseq data (GEO accession number: GSE120064)^14^. We used the broad cell type labels (cardiomyocytes, fibroblasts, etc.) and the subtype annotations (CM4, CM6, etc.) to deconvolve the spots. Differential expression analysis was conducted using Seurat “FindMarkers” function across conditions and cell types. Genes with absolute average log_2_ fold change > 1 and adjusted *P*-value (Benjamini-Hochberg method) < 0.05 were considered significantly differentially expressed. GSEA in GO terms was performed with clusterProfiler and org.Mm.eg.db R packages.

### Cell Culture

Cryopreserved adult human ventricular fibroblasts (HVFs) (Cell Applications, Inc) were cultured in Cardiac Fibroblast growth medium. Cells were subcultured using 0.25% trypsin when they reached 70% confluence and maintained at 37°C in a 5% CO_2_ humidified incubator. HVFs between the passages 2 to 6 were used for all *in vitro* experiments.

### CDCP1 Transient Knockdown

Cells were transfected with 25 nM siGENOME SMARTpool siRNAs (Dharmacon) targeting *CDCP1* using Lipofectamine RNAiMAX Reagent (ThermoFisher).

### Profibrotic Stimulation

HVFs were serum-starved overnight and treated with Recombinant human PDGF-BB (20 ng/ml), TGF-β (10 ng/ml), and Angiotensin II (100 ng/ml) in serum free DMEM. Reconstitution buffer was used as vehicle control. Depending on the experimental design, cells were harvested at 48 h, 72 h or 5 days post-stimulation for RNA extraction or immunofluorescence staining.

### Real-time Polymerase Chain Reaction (qRT-PCR)

Total RNA was extracted from HVFs using Quick-RNA MicroPrep Kit (Zymo Reseacrh). qRT-PCR was performed using a one-step Power SYBR Green RNA-to-CT kit (Applied Biosystems) on StepOne PCR system (Thermo Fisher). Ct values were normalized to the reference gene GAPDH, and the relative quantification was calculated using the ΔΔCt method.

### Immunofluorescence

HVFs were seeded in 96-well plates and cultured to 50% confluence prior to treatment with profibrotic stimuli. Following treatment, intact cells were either processed directly for immunofluorescence or subjected to decellularization to isolate cell-derived extracellular matrix (ECM). For intact-cell staining, nuclei were first labeled with Hoechst 33342 (1:1000) for 30 min at room temperature. For ECM preparation, cells were decellularized using a solution containing 20 mM ammonium hydroxide and 0.5% Triton X-100 for 5 min at room temperature. Both intact cells and decellularized ECM were gently washed three times with 1× PBS and fixed with 10% formalin for 10 min at room temperature. After fixation, samples were washed and blocked in 1× PBST (1% BSA, 0.1% Triton X-100) supplemented with 3% normal goat serum for at least 3 h or overnight at 4°C. Samples were then incubated overnight at 4°C with a rabbit anti–collagen I primary antibody (NB600–408G; 1:500). Following PBS washes, samples were incubated with Alexa Fluor 488–conjugated goat anti-rabbit secondary antibody (1:1000) together with DAPI (1:1000) for 1 h at room temperature. After final washes, collagen I immunofluorescence in both intact fibroblasts and decellularized ECM was imaged and quantified using a Cytation 5 imaging system.

### Statistical Considerations

Sample sizes for each experimental group were determined based on power calculations and anticipated effect sizes. All data are presented as mean ± standard deviation (SD). Two-way ANOVA (genotype · treatment) was used to assess main effects and interactions for echocardiographic and morphometric parameters across the four experimental groups. Pre-specified pairwise comparisons were performed using unpaired two-tailed Student’s *t*-tests. Two-way ANOVA with Sidak’s post hoc test was used for longitudinal body weight analysis. Survival analysis was performed using the log-rank (Mantel-Cox) test. For histological fibrosis quantification, an unpaired two-tailed Student’s *t*-test was applied. A *P*-value < 0.05 was considered statistically significant. GraphPad Prism (GraphPad Software, San Diego, CA) and R (v4.2.2) were used for statistical calculations and data plotting.

## RESULTS

### Generation and baseline characterization of Cdcp1 KO mice

**Figure 1:**
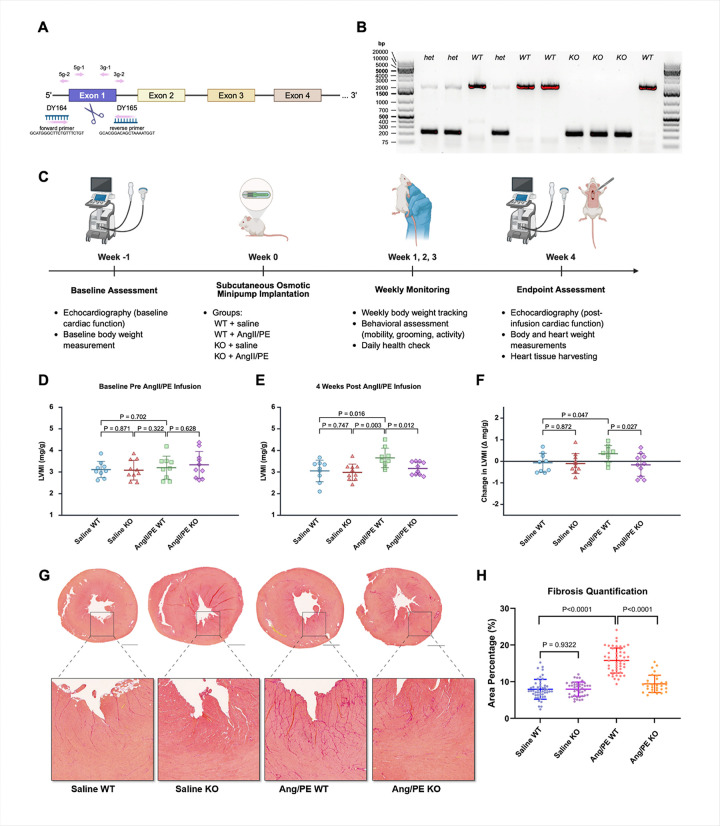
*Cdcp1* Deletion Attenuates Cardiac Hypertrophy and Fibrosis Induced by Pressure Overload. **A,** Schematic of CRISPR-Cas9–mediated *Cdcp1* knockout (KO) strategy. Four guide RNAs (5g-1, 5g-2, 3g-1, 3g-2) were designed to flank and excise exon 1. Genotyping primers (DY164, DY165) are indicated. **B,** PCR genotyping results confirming successful deletion of *Cdcp1*. Amplification yielded a 1,771 bp product for the wild-type (WT) allele and a truncated <501 bp product for the KO allele. **C**, Experimental timeline and procedures. At baseline (week −1), mice underwent transthoracic echocardiography and initial body weight measurement. At week 0, osmotic minipumps were subcutaneously implanted for chronic infusion of either saline or AngII/PE (1.2 μg/g/day AngII, 35 μg/g/day PE). Four experimental groups were studied. Animals were monitored weekly for body weight and general health. At the endpoint (week 4), cardiac function was reassessed by echocardiography, followed by euthanasia for collection of heart tissues and measurement of heart and body weights. **D**, Left ventricular mass index (LVMI, mg/g, calculated as LV mass divided by body weight) at baseline prior to AngII/PE infusion, **E**, LVMI at 4 weeks post AngII/PE infusion. **F**, Change in LVMI (Δ mg/g from baseline to 4 weeks). For D-F: Saline WT, n=9; Saline KO, n=10; AngII/PE WT, n=9; AngII/PE KO, n=10. Data presented as mean±SD. Statistical analysis was performed using two-way ANOVA (genotype ´ treatment) followed by pre-specified pairwise comparisons using unpaired two-tailed Student’s *t*-tests. **G**, Micrographs showing Picrosirius red-stained ventricular cross-sections from WT and *Cdcp1*-KO mice after 4 weeks of saline or AngII/PE infusion. Scale bars: 1 mm. **H**, Quantification of collagen-positive area (%) in each group (Saline WT, n=48; Saline KO, n=42; AngII/PE WT, n=48; AngII/PE KO, n=30). Data points represent individual fields with mean±SD overlay. Statistical significance was determined using unpaired Student’s *t*-test.

To investigate the role of *Cdcp1* in cardiac remodeling and fibrosis, we generated *Cdcp1*-KO mice using CRISPR-Cas9 editing to remove exon 1 of the *Cdcp1* gene (Fig. 1A). PCR genotyping confirmed the successful deletion of the targeted region by showing amplification products of 1,771 bp for the WT allele and a truncated product approximately 501 bp for the KO allele (Fig. 1B). Homozygous *Cdcp1*-KO mice were obtained at expected Mendelian ratios and were viable without overt congenital anomalies. To assess whether *Cdcp1* deletion affected baseline growth and development, we monitored body weight from weaning to adulthood. Longitudinal body weight measurements from 3 to 11 weeks of age showed comparable growth between WT (n = 14, 9 males, 5 females) and *Cdcp1*-KO mice (n = 10, 7 males, 3 females) (**Supplementary Fig. 1A**). Two-way ANOVA showed no significant main effect of genotype on body weight (*P* = 0.478) and no significant Age·Genotype interaction (*P* = 0.986). Sidak’s post hoc comparisons confirmed that there were no significant differences between genotypes at any time point (*P* > 0.05). To examine the effect of *Cdcp1* deletion on long-term survival during pressure overload, a separate cohort of mice was monitored for 14 weeks after osmotic minipump implantation (**Supplementary Fig. 1B**). All mice in the Saline WT (n = 9), Saline KO (n = 10), and AngII/PE KO (n = 9) groups had 100% survival throughout the observation period. In contrast, three deaths occurred in the AngII/PE WT group (n = 12), one at 13 weeks and two at 14 weeks post-implantation of AngII/PE infusion pump (log-rank test, *P* = 0.056). For chronic pressure overload, we implemented a 4-week experimental protocol (Fig. 1C). This timepoint captures the period of active fibrotic remodeling characteristic of the AngII/PE model, during which interstitial collagen accumulation precedes the onset of overt systolic dysfunction. Mice were monitored weekly for body weight and general health assessment. At the experimental endpoint, cardiac function was reassessed by echocardiography, followed by euthanasia for heart tissue collection and morphometric analysis. Collectively, these results demonstrated successful generation of viable *Cdcp1*-KO mice with normal baseline growth and development.

### Cdcp1 deletion attenuates cardiac hypertrophy and fibrosis after pressure overload

To investigate the impact of *Cdcp1* deletion on cardiac structure and function during pressure overload, we performed serial TTE at baseline and after 4 weeks of chronic AngII/PE infusion. At baseline, the left ventricular (LV) mass index (LVMI) was comparable across all groups (Fig. 1D). AngII/PE infusion induced significant cardiac hypertrophy in WT mice, with increased LVMI compared to saline-treated WT control (3.65 ± 0.45 mg/g vs. 3.05 ± 0.50 mg/g, *P* = 0.016) (Fig. 1E). This hypertrophic response was significantly attenuated in AngII/PE-treated *Cdcp1*-KO mice compared to AngII/PE-treated WT (3.17 ± 0.30 mg/g vs. 3.65 ± 0.45 mg/g, *P* = 0.012) (Fig. 1E), and *Cdcp1* deletion prevented the increase in LVMI from baseline observed in WT mice after pressure overload (−0.17 ± 0.53 mg/g vs. 0.35 ± 0.39 mg/g, *P* = 0.027) (Fig. 1F). Consistent with the 4-week timepoint capturing active fibrotic remodeling prior to systolic dysfunction, LV ejection fraction (LVEF) and LV fractional shortening (LVFS) remained within normal ranges across all groups, though AngII/PE-treated *Cdcp1*-KO mice showed trends toward higher values compared with AngII/PE-treated WT mice (**Supplementary Fig. 1C-D**). Histological analysis of Picrosirius red-stained heart sections revealed that AngII/PE treatment significantly increased collagen deposition in WT mice compared to saline controls, as expected (15.8 ± 0.5% vs. 7.9 ± 0.4%, *P* < 0.0001) (Fig. 1G). By contrast, this fibrotic response was markedly attenuated in *Cdcp1*-KO mice (9.4 ± 0.4% vs. 15.8 ± 0.5%, *P* < 0.0001), representing a 41% reduction in fibrotic burden (Fig. 1H). Saline-treated *Cdcp1*-KO and WT groups showed comparably low collagen-positive areas (7.6 ± 1.8% vs. 7.8 ± 2.2%), indicating no fibrotic changes due to *Cdcp1* deletion alone. Collectively, these findings demonstrate that *Cdcp1* deletion attenuates pressure overload-induced cardiac hypertrophy and fibrosis, the structural precursors to heart failure progression.

### RNA-seq identifies Cdcp1-dependent fibrotic and inflammatory gene programs in the left ventricle

**Figure 2:**
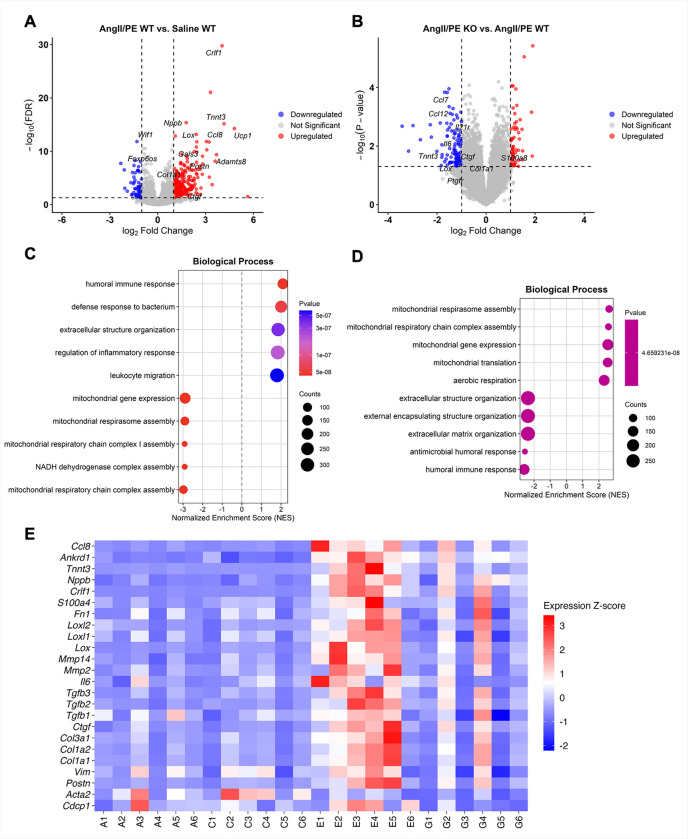
Transcriptomic Profiling Identifies *Cdcp1*-Dependent Pathways Driving Myocardial Fibrosis. **A,** Volcano plot of differentially expressed genes (DEGs) in WT mice treated with saline versus AngII/PE. **B,** Volcano plot of DEGs in *Cdcp1*-KO versus WT mice after AngII/PE treatment. **C,** GSEA of Gene Ontology (GO) Biological Process terms using ranked gene list ordered by log_2_ fold-change from the AngII/PE WT versus saline WT comparison. **D,** GSEA of GO Biological Process terms using a ranked gene list from the AngII/PE *Cdcp1-*KO versus AngII/PE WT comparison. For C and D, the top 5 positively and top 5 negatively enriched terms by adjusted P-value are shown. Dot size indicates gene set size; dot color indicates adjusted P-value. **E,** Heatmap showing expression profiles of extracellular matrix-related genes, inflammatory mediators, and selected markers (*Cdcp1, Tnnt3*).

To understand the molecular mechanisms underlying the effects of *Cdcp1* deletion, we performed RNA-seq using LV tissues from all experimental groups. Differential gene expression (DEG) analysis comparing AngII/PE-treated WT to saline-treated WT mice revealed 1,857 DEGs including 1,342 upregulated and 515 downregulated genes (Fig. 2A). There was significant upregulation of cardiac stress markers (*Nppb*, *Tnnt3*), pro-fibrotic and ECM remodeling factors (*Lox*, *Postn*, *Col1a1*, *Adamts8*), and inflammatory mediators (*Ccl8*, *Gals3*), which is consistent with the pathological remodeling typically observed in pressure overload. No transcriptional differences were found when comparing saline-treated *Cdcp1*-KO and WT mice, suggesting that *Cdcp1* deletion alone has no effect on baseline cardiac gene programs (**Supplementary Fig. 2**). However, when we compared AngII/PE-treated *Cdcp1*-KO with AngII/PE-treated WT mice, we found 783 DEGs including 424 downregulated and 359 upregulated genes (Fig. 2B). *Cdcp1* deletion in the context of pressure overload was associated with significant downregulation of pro-fibrotic factors (*Ctgf*, *Lox*, *Col1a1*) and inflammatory mediators (*Ccl7*, *Ccl12*, *Il21r*, *Il6*). Gene set enrichment analysis (GSEA) of GO Biological Process terms using a ranked gene list ordered by log_2_ fold-change identified extracellular structure organization, regulation of inflammatory response, and leukocyte migration among the most significantly positively enriched pathways in AngII/PE-treated versus saline-treated WT mice, consistent with the observed cardiac remodeling (Fig. 2C). When the same analysis was applied to the AngII/PE-treated *Cdcp1*-KO versus WT mice, extracellular matrix organization, external encapsulating structure organization, and immune-related terms were negatively enriched, while mitochondrial gene expression and respiration pathways were positively enriched (Fig. 2D), suggesting that CDCP1 may also be involved in regulating immune responses in pressure-overloaded hearts. Furthermore, AngII/PE treatment markedly increased expression of ECM genes (*Col1a1*, *Col1a2*, *Col3a1*), fibroblast activation markers (*Vim*, *Ctgf*), matrix remodeling enzymes (*Mmp2*, *Mmp14*), inflammatory chemokines (*Ccl7*, *Ccl12*), the cardiac-stress marker *Tnnt3*, and *Cdcp1* itself in WT heart LVs, whereas the increase in expression of each of these genes was significantly attenuated in *Cdcp1*-KO heart LVs (Fig. 2E).

### Spatial Transcriptomics Reveals Region- and “Cell Type”-Specific Remodeling Suppressed by Cdcp1 KO in Pressure Overload

**Figure 3:**
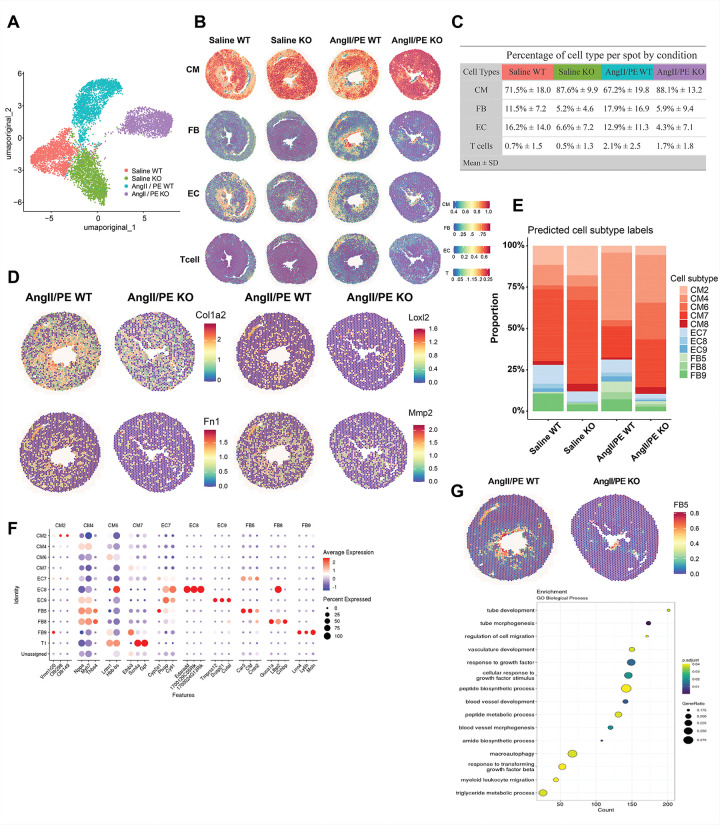
Spatial transcriptomic analysis reveals *Cdcp1*-dependent alterations in cardiac cell-type composition under pressure overload. **A**, UMAP embedding of spatial transcriptomic spots calculated on the first 30 principal components prior to integration, colored by sample of origin.**B,** Global spatial deconvolution mapping the distribution of identified cell types across heart tissue sections (Cardiomyocytes: CM; fibroblasts: FB; endothelial cells: EC; T-cells). Color scale indicates inferred proportion of spot corresponding to the cell type. **C,** Table showing mean ± SD of deconvoluted cell type proportions per spot across all four groups. **D,** Spatial dot plots showing expression of extracellular matrix-associated genes (*Col1a2, Fn1, Mmp2, Loxl1*) **E,** Relative proportions of major cell subtype populations across saline WT, saline KO, AngII/PE WT, and AngII/PE KO samples. **F,** Dot plot of marker gene expression used to define cardiomyocyte, fibroblast, and endothelial subpopulations. **G,** Spatial distribution and GSEA for GO biological process of FB5 fibroblast subpopulation in AngII/PE WT versus KO spots.

To investigate the spatial distribution of transcriptional changes associated with *Cdcp1* deletion during pressure overload, we performed spatial transcriptomic profiling of cardiac sections from all four experimental groups (**Supplementary Fig. 3**). Across all samples, we captured a total of 7,889 spatial barcoded transcriptomic spots (Saline_WT: 1,857; Saline_KO: 1,915; AngII/PE_WT: 2,193; AngII/PE_KO: 1,924), with median gene counts per spot ranging from 2,049 to 3,710. UMAP embedding calculated on the first 30 principal components showed distinct separation of spots according to sample of origin before integration (Fig. 3A), reflecting systematic transcriptomic differences across the samples. Using a reference-guided deconvolution approach, we inferred the relative contributions of major cardiac cell types, across the myocardial sections, including cardiomyocytes, fibroblasts, endothelial cells, and immune cells (Fig. 3B). Cardiomyocytes were the predominant cell type across all groups (67.2–88.1%), followed by fibroblasts (5.2–17.9%), endothelial cells (4.3–16.2%), and T cells (0.5–2.1%). Quantification of cell-type proportions suggested a consistent reduction in fibroblast abundance in *Cdcp1*-KO hearts compared with WT controls, observed both under saline conditions and following AngII/PE treatment. While AngII/PE-treated WT hearts exhibited a marked expansion of predicted fibroblasts (18%), this increase was entirely blunted in *Cdcp1*-KO hearts (6%) (Fig. 3C). Together, these findings indicate that *Cdcp1* deletion is associated with reduced fibroblast representation in the myocardium particularly in the context of pressure overload. Consistent with these compositional changes, spatial expression mapping of extracellular matrix–associated genes revealed reduced fibrogenic signaling in *Cdcp1*-deficient hearts. Expression of *Col1a2, Fn1, Mmp2*, and *Loxl2* were prominently enriched in AngII/PE-treated WT hearts but substantially diminished in AngII/PE-treated *Cdcp1*-KO hearts (Fig. 3D), indicating suppression of ECM production and matrix remodeling transcriptional programs in the absence of *Cdcp1*.

To further resolve cellular heterogeneity, we next examined subpopulation structure within major cardiac cell types. Deconvolution analysis identified multiple transcriptionally distinct subclusters of cardiomyocytes, fibroblasts, and endothelial cells, whose relative proportions differed across experimental groups (Fig. 3E). Marker gene analysis confirmed robust and cell-type–specific expression patterns defining each subcluster (Fig. 3F). Five distinct cardiomyocyte subtypes (CM2, CM4, CM6, CM7, and CM8) were identified across all experimental groups (**Supplementary Fig. 4A**). Quantitative analysis revealed that AngII/PE-treated WT hearts exhibited a relative enrichment of the CM4 cardiomyocyte subtype, which was enriched for inflammatory response, extracellular matrix organization, collagen fibril organization, and wound healing pathways (**Supplementary Fig. 6A**). In contrast, this cell type population was reduced in AngII/PE-treated *Cdcp1*-KO hearts, whereas the CM6 subtype, which was enriched for muscle cell differentiation, myofibril assembly, cell-substrate adhesion, and integrin-mediated signaling pathways (**Supplementary Fig. 6B**), was increased in *Cdcp1*-KO hearts compared with AngII/PE-treated WT (**Supplementary Fig. 4B**).

Similarly, deconvolution of fibroblast and endothelial compartments identified multiple transcriptionally distinct subtypes whose proportions differed across experimental conditions (**Supplementary Fig. 4A**). Notably, all the three profibrotic fibroblast subtypes (FB5, FB8, and FB9) were expanded in AngII/PE-treated WT hearts but attenuated in *Cdcp1*-KO hearts, while endothelial subtypes showed more modest but consistent shifts in abundance (**Supplementary Fig. 4B**). Among fibroblast subpopulations, FB5 showed the most prominent *Cdcp1*-dependent differences. Gene set enrichment analysis revealed that FB5 was enriched for biological processes related to response to growth factors, response to TGF-β, regulation of cell migration, and vasculature development (Fig. 3G). Notably, FB5 exhibited high expression of collagen and ECM-associated genes, consistent with a profibrotic fibroblast state **(Supplementary Fig. 4C)**. This subpopulation was prominently expanded in AngII/PE-treated WT compared to *Cdcp1*-KO hearts, linking the presence of *Cdcp1* expression to the emergence of collagen-producing fibroblast niches during pressure overload

**In summary**, these analyses demonstrate *Cdcp1* deletion attenuates pathological cardiac remodeling, reduces histological fibrosis, and is associated with diminished fibroblast expansion and ECM gene expression. These results are consistent with our prior demonstration that *Cdcp1* contributes to cytokine driven expansion of cardiac fibroblasts.^11^ To determine whether CDCP1 directly influences human fibroblast fibrogenic programs, we next examined CDCP1-dependent extracellular matrix programs in human ventricular fibroblasts.

### CDCP1 regulates growth factor-induced extracellular matrix related gene expression and collagen deposition in human ventricular fibroblasts

**Figure 4:**
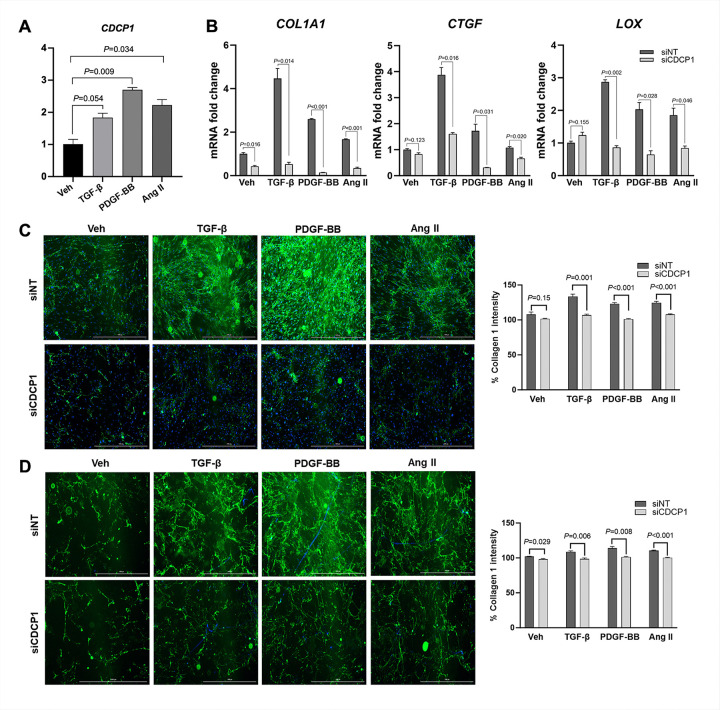
*Cdcp1* deficiency attenuates profibrotic gene expression and collagen deposition in human ventricular fibroblasts. HVFs were treated with profibrotic stimuli PDGF-BB (20 ng/mL), TGF-β (10 ng/mL), or angiotensin II (100 ng/mL), individually for 48 h. **A,** *CDCP1* expression was assessed by qRT-PCR. **B,** mRNA levels of extracellular matrix-associated genes (*COL1A1, CTGF, LOX)* after CDCP1 knockdown. *GAPDH* was used as internal control. **C,** Representative immunofluorescence images of collagen I in intact HVFs following profibrotic stimulation upon CDCP1 knockdown. **D,** Immunofluorescence staining of collagen I in decellularized fibroblast-derived extracellular matrix.

To directly test fibroblast fibrogenic responses, we examined the effects of *CDCP1* knockdown in human ventricular fibroblasts (HVFs) exposed to profibrotic stimuli. HVFs were treated with platelet-derived growth factor-BB (PDGF-BB), transforming growth factor-β (TGF-β), and angiotensin II, all of which are key signaling pathways enriched in FB5 fibroblasts *in vivo*. Stimulation with individual fibrotic cues led to a significant induction of *CDCP1* expression (Fig. 4A), suggesting *CDCP1* as a fibroblast-intrinsic, stress-responsive gene involved in profibrotic signaling pathways. Silencing of *CDCP1* markedly attenuated the induction of extracellular matrix–associated genes across all fibrotic stimuli, including *COL1A1*, *CTGF*, and *LOX* (Fig. 4B). These genes represent core components of collagen production, matrix cross-linking, and fibroblast activation, consistent with the transcriptional programs enriched in FB5 fibroblasts identified in spatial transcriptomic analyses.

At the protein level, immunofluorescent staining of intact HVFs demonstrated a robust reduction in collagen I production following *CDCP1* knockdown under profibrotic stimulation (Fig. 4C). Furthermore, analysis of decellularized extracellular matrix confirmed a corresponding decrease in collagen I deposition (Fig. 4D), indicating that *CDCP1* regulates not only fibroblast gene expression but also synthesis and deposition of collagen I, the predominant component of fibrotic scar in the heart, by activated fibroblasts. Together, these findings establish CDCP1 as a fibroblast-intrinsic regulator of growth factor driven extracellular matrix gene expression and collagen deposition, providing a mechanistic link to the attenuated fibrotic remodeling seen in *Cdcp1*-deficient hearts.

## DISCUSSION

In this study, we demonstrate that CDCP1 is a regulator of extracellular matrix production in cardiac fibroblasts. Global *Cdcp1* deletion attenuates pressure overload-induced cardiac hypertrophy, collagen deposition, suppresses ECM gene expression, and reduces pro-fibrotic fibroblast subpopulations, a finding that is supported by *CDCP1* knockdown in human cardiac fibroblasts.

Consistent with prior GWAS findings linking lower *CDCP1* expressions to improved cardiac function in patients with heart failure^11,12^, we found that *Cdcp1* KO attenuated pressure overload-induced increases in LV mass, whereas LVEF and LVFS showed a trend toward improved systolic function. This is consistent with early fibrotic remodeling preceding systolic dysfunction^15^ in this model.^16,17^ *Cdcp1* KO mice exhibited significantly reduced collagen deposition compared to wild-type controls in a pressure overload model, with no evidence of baseline fibrosis in saline-treated hearts, indicating that *CDCP1* is dispensable under physiological conditions but contributes to maladaptive remodeling during cardiac stress. Bulk RNA sequencing revealed that *Cdcp1* deletion suppressed the expression of a broad array of fibrosis-associated genes, including *Col1a1*, *Postn*, *Lox*, and *Ctgf*, as well as inflammatory chemokines such as *Ccl7*, *Ccl12*, and *Il6*. These results suggest that *Cdcp1* may act upstream of canonical fibrotic and inflammatory gene programs, potentially through modulation of fibroblast activation and ECM remodeling pathways.

Mechanistically, our prior *in vitro* studies demonstrated that *CDCP1* is upregulated in human cardiac fibroblasts in response to PDGF-BB stimulation and that *CDCP1* knockdown suppresses fibroblast proliferation via reduced AKT phosphorylation.^11^ The present *in vivo* findings, specifically the reduced fibroblast abundance observed in *Cdcp1*-KO hearts by spatial transcriptomics, are consistent with this proliferation-dependent mechanism. PDGFRα is uniquely expressed by cardiac fibroblasts and is essential for their survival through PI3K signaling. PDGFRα loss causes approximately 50% reduction in resident cardiac fibroblasts within days.^18^ Importantly, controlled reduction of PDGFRα^+^ fibroblasts (60–80%) has been shown to preserve cardiac function following AngII/PE-induced cardiac fibrosis,^19^ supporting the therapeutic potential of targeting this pathway. Our prior data suggests that *CDCP1* functions as a modulator of PDGF-AKT signaling in cardiac fibroblasts, and its deletion may attenuate sustained AKT activation required for fibroblast expansion and survival.

To further define the spatial and cell-type–specific impact of *Cdcp1* deletion, we leveraged spatial transcriptomics. Deconvolution analysis showed that *Cdcp1*-KO hearts had reduced fibroblast abundance and altered spatial distribution under pressure overload. Importantly, *Cdcp1* deletion led to a marked reduction of the FB5 and FB8 fibroblast subtypes, which were associated with TGF-β signaling, growth factor responses, vascular remodeling, and migration. The transcriptional profile of FB5 – enriched in ECM organization, growth factor signaling, and localized to interstitial and perivascular regions – corresponds to the THBS4+/CILP + pro-fibrotic fibroblast populations recently characterized in pressure overload models.^20,21^ These populations, which express *Thbs4*, *Cilp*, *Postn*, and *Cthrc1*, represent a pressure overload-specific fibroblast state distinct from classical α-SMA^+^ myofibroblasts observed after myocardial infarction.^22^ Our prior findings suggest that *CDCP1* plays a role in the emergence or maintenance of these ECM-producing fibroblast populations through AKT-mediated survival signaling.^11^

Cardiomyocyte subpopulation analysis revealed more layers of *Cdcp1*-dependent remodeling. In WT hearts under pressure overload, the emergence of CM4 – a pro-fibrotic cardiomyocyte subtype enriched in Wnt signaling, ECM remodeling, and inflammatory gene signatures – was pronounced. However, this population was significantly reduced in *Cdcp1*-KO hearts. Instead, *Cdcp1*-deficient hearts displayed enrichment of CM6, a cardiomyocyte subtype characterized by signatures of muscle cell development, cell-substrate adhesion, and myofibril assembly, suggesting a potentially reparative or adaptive remodeling phenotype. The spatial co-localization of CM4 with FB5 clusters in WT hearts, (**Supplementary Figs. 4 and 5**) and the coordinated reduction of both populations in *Cdcp1*-KO hearts, suggests that *CDCP1* may regulate pathological fibroblast-cardiomyocyte crosstalk during pressure overload.

Our findings place *CDCP1* within a growing class of transmembrane proteins validated as cardiac fibrosis targets. For instance, fibroblast activation protein (FAP) emerged as a promising therapeutic target. FAP-specific CAR T cells have been shown to reduce fibrosis and improve function in the AngII/PE model,^23^ and more recently, *in vivo* generation of transient FAP-targeted CAR T cells via CD5-targeted lipid nanoparticle (LNP)-mediated delivery of modified mRNA enabled transient, controllable intervention.^24^ Depletion of αv integrin in PDGFRβ^+^ cells confers protection against cardiac fibrosis, and small molecule inhibitors similarly attenuate established fibrosis via the shared SRC-PI3K/AKT pathway.^25^ IL-11 receptor (IL-11RA) KO mice are protected from cardiac fibrosis, and neutralizing antibodies demonstrate preclinical efficacy against it.^26^ Like these targets, *CDCP1* is a cell-surface transmembrane protein with limited cardiomyocyte expression, rendering it suitable for antibody-based therapeutic strategies currently under development in oncology.^27,28^

Importantly, *CDCP1* may have context-dependent roles across different fibrotic pathways, cells and organs. In lung fibroblasts, *CDCP1* knockdown enhanced TGF-β1-induced myofibroblast differentiation,^29^ in contrast to our findings in cardiac fibroblasts, where *CDCP1* promotes PDGF-driven proliferation and is necessary for TGF-β mediated ECM synthesis. This organ specific function warrants further mechanistic investigation.

Several limitations of this study should be acknowledged. First, the use of a global Cdcp1 knockout mouse model precludes definitive attribution of the observed phenotypes to specific cell types; although convergent in vivo and in vitro data implicate fibroblasts, cell type–specific deletion (e.g., fibroblast- or cardiomyocyte-restricted models) will be necessary to delineate causal mechanisms and intercellular contributions more precisely. Notably, however, the use of a global knockout may also enhance translational relevance, as a pharmacologic strategy targeting CDCP1 would likely exert systemic effects rather than cell type–restricted modulation. Second, the AngII/phenylephrine infusion model recapitulates aspects of pressure overload–induced remodeling but does not fully capture the heterogeneity of human heart failure etiologies, limiting direct translational generalizability. Third, the primary analyses were conducted at an early time point (4 weeks), when fibrotic remodeling precedes overt systolic dysfunction, and therefore do not establish long-term functional consequences or effects on advanced heart failure phenotypes. Fourth, spatial transcriptomic resolution is limited by spot size and reliance on computational deconvolution, which may obscure finer cellular heterogeneity and introduce inference bias in cell-type assignments. Fifth, while human ventricular fibroblast experiments support a conserved, fibroblast-intrinsic role for CDCP1, these reductionist systems do not fully recapitulate the multicellular myocardial environment or systemic influences present in vivo. Finally, although CDCP1 emerges as a potential therapeutic target, the safety, tissue specificity, and efficacy of CDCP1-directed interventions—particularly given its context-dependent roles across organs—remain to be established in translational and large-animal studies.

From a translational perspective, *CDCP1* is a promising therapeutic target for cardiac fibrosis. As a transmembrane protein with an accessible extracellular domain, it is amenable to antibody-based therapies already in development for oncologic indications,^27,28^ which could potentially be repurposed for this indication. Moreover, *Cdcp1* deletion is well-tolerated at baseline and elicits pathological phenotypes only under stress, suggesting a favorable therapeutic window. Human genetic data from the UK Biobank PheWAS link *CDCP1* variants to heart failure mortality,^11^ and conserved mechanisms in human cardiac fibroblasts reinforce its translational potential.

In conclusion, our study provides direct *in vivo* evidence that *CDCP1* attenuates pressure overload-induced cardiac fibrosis. By integrating genetic, histological, and spatial transcriptomic approaches, we demonstrate that *Cdcp1* deletion suppresses pro-fibrotic THBS4^+^/CILP^+^-like fibroblast populations, attenuates pathological cardiomyocyte states, and preserves cardiac structure. Mechanistically, *CDCP1* functions as an important mediator of multiple pro-fibrotic stimuli that drive fibroblast collagen synthesis. These insights provide a mechanistic basis for targeting *CDCP1* to attenuate cardiac fibrosis and thus heart failure progression.

## Supplementary Material

Supplementary Files

This is a list of supplementary files associated with this preprint. Click to download.

• SUPPLEMENTARYFIGURESlegends.docx

• SupplementaryFigure4.png

• SupplementaryFigure2.tif

• SupplementaryFigure5.png

• SupplementaryFigure6.tif

• SupplementaryFigure1.tif

• SupplementaryFigure3.tif

• FullUncroppedGel.pdf

## Data Availability

The bulk RNA sequencing data generated during this study are available in the NCBI Gene Expression Omnibus (GEO) repository under accession number GSE296565. The spatial transcriptomics data is available under accession number GSE303631. All analysis scripts and computational pipelines used in this study are publicly available at https://github.com/irenemaring/CDCP1_PressureOverload. No custom algorithms were developed. All code for standardized analyses (Seurat, DESeq2,) is publicly accessible through GitHub or CRAN/Bioconductor repositories.
